# A rare case of infectious colitis

**DOI:** 10.1093/gastro/gov016

**Published:** 2015-05-25

**Authors:** Aditya Kalakonda, Shashank Garg, Suraj Tandon, Rakesh Vinayak, Sudhir Dutta

**Affiliations:** ^1^State University of New York (SUNY) Upstate Medical University, Syracuse, NY, USA; ^2^Department of Gastroenterology, Sinai Hospital of Baltimore, Baltimore, MD, USA

**Keywords:** methicillin-resistant *Staphylococcus aureus*, infectious colitis, stool culture

## Abstract

Methicillin-resistant *Staphylococcus aureus* (MRSA) is responsible for numerous infectious processes. Gastrointestinal tract involvement is rather rare and only a handful of cases of MRSA colitis have been reported in North America. We present a case of MRSA colitis in an adult without apparent risk factors. Abdominal computed tomography (CT) showed thickening of the sigmoid colon, indicative of colitis, and empiric therapy with ciprofloxacin and metronidazole was started. Initial work-up for infection—including blood and stool cultures, and stool *Clostridium difficile* toxin assay—was negative. The patient’s clinical status improved but his diarrhea did not abate. Repetition of stool culture demonstrated luxuriant growth of MRSA sensitive to vancomycin. Oral vancomycin was administered and the patient’s symptoms promptly ceased.

## Introduction

Enterocolitis secondary to methicillin-sensitive *Staphylococcus aureus* (MSSA) has been described since the 1950s and has typically been associated with peri-operative antibiotic therapy and gastrointestinal surgery [1]. In recent years its prevalence has been re-assessed given the isolation of other pathogens, mainly *Clostridium difficile* (*C. difficile*) [2]*.* Although MSSA has been frequently isolated, methicillin-resistant *Staphylococcus aureus* (MRSA) has, in rare cases, been identified as the cause of colitis. MRSA colitis has seldom been reported in North America, with only a very small number of documented cases. We present a rare case of MRSA colitis in an adult without significant risk factors or history of gastroenterological illness.

## Case presentation

A 34-year-old Caucasian male was brought to the emergency room following multiple episodes of non-bilious vomiting and non-bloody diarrhea over the previous three days. This was associated with abdominal bloating, listlessness, decreased oral intake and decreased urine output. The patient's history was obtained from his mother as the patient was unable to speak. She did not notice any fever, chills, or sweating. She did not report any recent hospitalization or sick contacts for the patient. His past medical history was significant for developmental delay, club foot, neural deafness and type II diabetes. Family history was unremarkable in terms of any infectious or gastrointestinal illnesses. His mother was his primary carer. The patient did not have a history of alcohol, tobacco or illicit drug use. He was not taking any medications at home and did not have any reported allergies.

Vital signs in the emergency room were significant for a temperature of 38.7°C, heart rate of 153 bpm, blood pressure of 74/43 mmHg, respiratory rate of 55 with 90% oxygen saturation on 2 L of supplemental oxygen via nasal cannula. General examination was remarkable for lethargy, dry mucous membranes, collapsed neck veins, and decreased skin turgor. Abdominal examination revealed a distended abdomen with marked left lower quadrant tenderness. No guarding or rebound tenderness was noted. Bowel sounds were remarkably decreased. Laboratory analysis showed a white blood cell count of 20.9 cells/mL with 10% band neutrophils and 62% mature neutrophils, blood urea nitrogen 57 mg/dL, creatinine 2.75 mg/dL, bicarbonate 13 mmol/L with an anion gap of 26 and lactic acid level of 10.3 mmol/L. Liver enzymes, total bilirubin and coagulation factors were within normal limits. Work-up for sources of infection included blood cultures, stool culture, stool *C. difficile* toxin polymerase chain reaction (PCR) and urine analysis.

**Figure 1.**
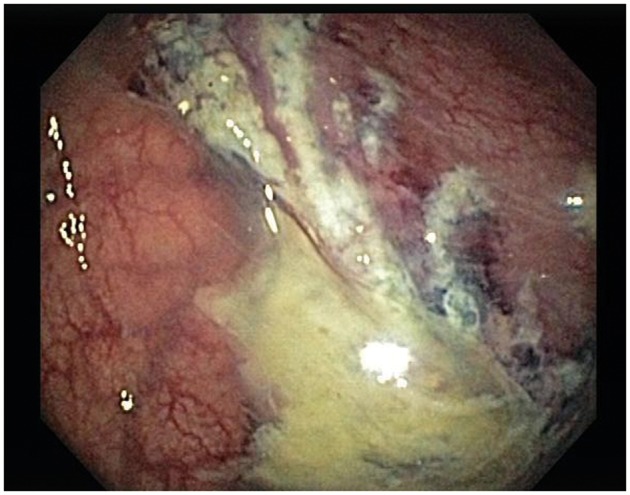
Endoscopic findings

The patient was started on intravenous fluid therapy for dehydration, along with ciprofloxacin and metronidazole for suspected infectious colitis. Abdominal plain films showed a distended colon, suggestive of distal colonic obstruction *vs.* intestinal ileus. Computed tomography (CT) scan of the abdomen showed thickening of the sigmoid colon with distension of the transverse and descending colon, consistent with colitis. The patient underwent a colonoscopy, which showed severe inflammation with mucosal friability and grey-white exudates extending from the sigmoid to the descending colon (Figure 1). A sudden transition to fairly normal-looking colon was noted in the transverse colon. Multiple biopsies were taken from the affected and seemingly normal areas.

The patient’s hypotension and lactic acidosis resolved with intravenous fluids and creatinine trended down to normal by hospital day 5, but his diarrhea did not abate. Stool culture was negative for Salmonella, Shigella, Campylobacter, and Yersinia. Blood cultures, urine analysis and stool *C. difficile* toxin assay were also negative. Colonoscopic biopsies showed mild active colitis with fibrino-purulent exudates. Given the negative work-up for infectious colitis and active inflammation of pathology, the patient was started on intravenous corticosteroids and mesalamine rectal enemas for proctitis from suspected ulcerative colitis. Additionally, a repeat stool specimen was sent for culture. On the sixth day following admission, the repeat stool culture came back positive for heavy growth of MRSA. The patient was subsequently started on vancomycin 125 mg orally QID for 14 days. After the institution of vancomycin, the patient's symptoms promptly resolved. He was taken off the corticosteroids. His repeat stool culture 30 days later was negative for MRSA, indicating cure.

## Discussion

*Staphylococcus aureus* is a normal inhabitant of the gastrointestinal tract and can be cultured in many individuals as part of normal bowel flora. In the mid-twentieth century, pseudomembranous enterocolitis, secondary to MSSA, increased in prevalence among patients who received either peri-operative antibiotic therapy or who underwent major gastrointestinal surgery [1–3]. MSSA was implicated as the causative agent based on positive stool cultures and enterotoxin studies; however, these diagnoses were made prior to isolation of the *C. difficile* enterotoxin. Since its isolation in 1978 among patients identified with pseudomembranous enterocolitis, the diagnosis rate of MSSA enterocolitis had sharply decreased [3, 4].

Since its discovery in 1961 MRSA, like MSSA, has become an increasingly common pathogen in the nosocomial and community environment. In the United States it is estimated that on average 94 000 patients annually are infected with MRSA, resulting in 19 000 deaths [5]. The primary disease process associated with community-acquired invasive MRSA has been reported to be bacteremia (75.2%), pneumonia (13.3%), cellulitis (9.7%), osteomyelitis (7.5%), endocarditis (6.3%), and septic shock (4.3%) [5]. The nares are considered to be the primary carriage source for MRSA; however, the intestine and rectum have been estimated to have colonization rates as high as 10% [6] and 60% [4], respectively. Although MRSA is prevalent in the community, it rarely poses a risk to healthy individuals. Unlike MSSA, MRSA has not been frequently isolated as a cause of colitis.

MRSA colitis has been documented in a small subset of individuals in Asian literature [7]. No similar extent of cases within similar patient populations has been reported in western literature. To the best of our knowledge, we present the sixth case of MRSA colitis in the western literature (Table 1) [8–12]. In published data, there seems to be some relationship between recent antibiotic use, acid-suppressive therapy and recent abdominal surgery, and the development of MRSA colitis [13, 14]. Specifically, MRSA colitis has been found to occur most commonly in patients who have undergone recent gastric surgery. Generally these patients developed MRSA colitis two to seven days after surgery. It has been postulated that the initiation of broad-spectrum antibiotics, elevation of gastric pH juices due to anti-peptic ulcer drugs, and lack of peristalsis all contribute to the development of MRSA-related colitis in these patients [6]. As witnessed in our patient, MRSA can cause spontaneous, invasive, gastrointestinal disease despite the absence of any risk factors.

**Table 1:** Review of reported cases of MRSA colitis in the western literature

Case	Age	Sex	Presentation	Risk factors	Laboratory evaluation	Radiographic / endoscopic findings	Diagnostic method	Treatment
Taylor *et al.* [8], 1993	71	M	Diarrhea, pruritis, reduced urine output	Crohn’s disease, history of previous right hemicolectomy	WBC: 11.2x10^9^/L; Cr: 747 mol/L	Not available	Stool culture	Oral vancomycin x 8 days. No follow-up stool culture
Schiller *et al.* [9], 1998.	64	F	Nausea, vomiting, watery diarrhea x 1 week	Remote splenectomy and hemigastrectomy, Klebsiella pneumonia treated with multiple antibiotics 1 week prior to symptom onset	Not available	Sigmoidoscopy: patchy sigmoid colitis	Stool culture	Oral vancomycin: unknown duration. No follow-up stool culture
McPherson *et al.* [10], 2005	43	F	Watery diarrhea, colicky abdominal pain, vomiting x 1 day	Hysterectomy and prophylactic antibiotics 1 day prior to symptom onset, healthcare worker	WBC: 18 000/mm^3^; CRP: 102 mg/L	X-ray of abdomen: dilated loops of bowel; Sigmoidoscopy: normal	Stool culture	Oral vancomycin x 10 days. No follow-up stool culture
Cheng *et al.* [11], 2006	39	M	High output ileostomy, x 1 day	Appendectomy and prophylactic antibiotics 9 days and Hartmann’s procedure 5 days prior to symptom onset	WBC: 14 800/mm^3^; CRP: 99 mg/L	Not available	Stool culture	IV vancomycin: unknown duration. Follow-up stool culture showed MRSA colonization
Clarke *et al.* [12], 2012	60	F	Diarrhea, fever, bright red blood *per rectum*, x 4 weeks	Health care worker	CRP: 14 times upper limit of normal	CT of abdomen: pan colitis; Colonoscopy: pan colitis with ulcerations in transverse colon	Stool culture	Oral vancomycin: unknown duration. No follow-up stool culture
Current case	34	M	Diarrhea, vomiting, abdominal distension x 3 days	None identified	WBC: 20 900/mm^3^; BUN:57 mg/dL; Cr: 27.5 mg/L; CO_2_: 13 mmol/L; Lactic acid: 10.3 mmol/L	X-ray of abdomen: colonic distension; CT of abdomen: left-sided colitis; Colonoscopy: pseudomembranous colitis	Stool culture	Oral vancomycin x 14 days. Follow-up stool culture negative for MRSA

BUN = blood urea nitrogen; Cr = creatinine; CRP = C-reactive protein; F = Female; M = Male; MRSA = methicillin-resistant *Staphylococcus aureus*; WBC = white blood count

MRSA colitis is characterized by high fever, abdominal distension and watery diarrhea that often leads to severe dehydration, shock, a sharp increase in white cell counts and sometimes multi-organ failure. In patients with hospital-acquired diarrhea of unknown etiology, risk factors such as hemigastrectomy and broad spectrum antibiotic use should alert the physician to considering the possibility of staphylococcal enterocolitis [9, 13, 14]. The reported literature suggests that stool gram stain and culture is the mainstay of the diagnosis. Imaging studies and colonoscopy can help in ruling out other disorders or confirming the diagnosis. Oral vancomycin has been the mainstay of therapy for MSSA enterocolitis and using a similar regimen for 10–14 days in MRSA colitis also seems to be an effective treatment modality (Table 1). Although vancomycin-resistant *Staphylococcus aureus* has been increasing in prevalence [15], there have been no reported cases of resistance to vancomycin in MRSA colitis.

It should also be mentioned that it is equally likely that our patient may have developed MRSA colitis as a result of initiation of empiric broad-spectrum antibiotic therapy. This could have resulted in an imbalance in normal colonic flora, leading to an excess growth of MRSA. Given its complications, physicians should be aware of its rapid clinical course and initiate prompt treatment with vancomycin to prevent decline in a patient's clinical course. Unrecognized and untreated MRSA colitis may pose a significant strain on the patient's hospital course, while incurring additional hospital costs.

*Conflict of interest statement*: none declared.
